# Circular RNAs as disease modifiers of complex neurologic disorders

**DOI:** 10.3389/fphar.2025.1577496

**Published:** 2025-05-16

**Authors:** Veronica Galli, Sara Vinciguerra, Marta Biagioli, Jasmin Morandell

**Affiliations:** NeuroEpigenetics Laboratory, Department of Cellular, Computational and Integrative Biology - CIBIO, University of Trento, Trento, Italy

**Keywords:** circRNA, non-coding RNA, alternative splicing, neurodevelopment, neurodegeneration

## Abstract

Circular RNAs are a large class of non-coding RNA molecules, conserved across species and produced by back-splicing. While their molecular functions are still elusive, the ones primarily retained in the nucleus are usually associated to regulation of transcription and mRNA processing patterns. Instead, the majority, are transported to the cytoplasm where they elicit micro-RNA (miRNA) or RNA binding protein (RBP)-sponging functions, or could be translated. CircRNAs are abundantly expressed in brain tissue, where they do not only act as regulators of brain development and physiology, but can also contribute to complex neurological conditions. In fact, deregulated circRNA expression levels were described in neurodevelopmental and neurodegenerative disorders, like Alzheimer’s disease, Parkinson’s disease and Huntington’s disease. Because of their described roles in pathology, these molecules may not only represent possible disease bio-markers, but they could even function as disease modifiers. As such, they could be targeted or protected in search of novel routes of therapeutic intervention. In this review, we highlight recent developments in the field, first discussing circRNAs involved in physiologic brain development and function, then reviewing studies that implicate circRNAs in neurodevelopmental and neurodegenerative disorders, with major attention to experimental studies exploring circRNA function and their role in neuropathologic processes. Such experimental strategies are mainly based on depletion or over-expression approaches and provide important insights into the modulatory potential of these molecules. They are relevant for clinical translation of basic research findings to drug development, possibly generating a positive impact for patients’ quality of life.

## Introduction

**FIGURE 1:**
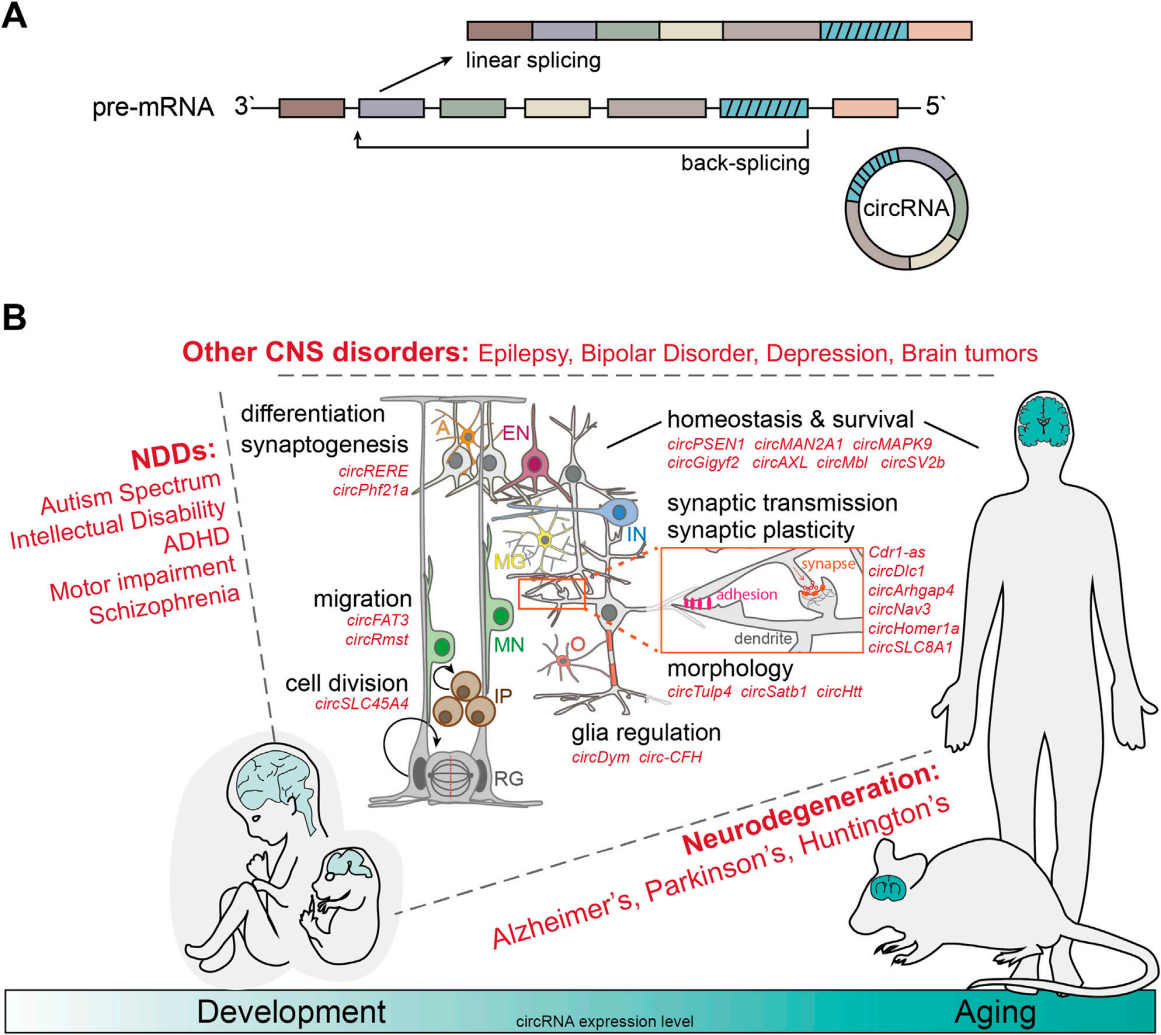
CircRNAs as modifiers of complex brain disorders. **(A)** Schematic of linear splicing (top) vs back-splicing (bottom). Back-splicing leads to the formation of a covalent bond between the 3′-splice donor and a 5′-splice acceptor site of unprocessed pre-mRNAs, generating the unique back-splicing sequence and rendering the resulting circRNAs extremely resistant against degradation through exoribonucleases. **(B)** CircRNAs are evolutionarily conserved and especially abundant in the nervous system of a large number of different species and especially mammals. Globally, circRNA expression increases during brain development, maturation and aging (bottom). In the last decade, numerous studies have linked deregulation of a variety of circRNAs with both, neurodevelopmental disorders (NDDs) such as Autism Spectrum Disorders and Schizophrenia (left); neurodegenerative (NDG) processes such as Alzheimer’s, Parkinson’s and Huntington’s Disease, as well as Frontotemporal dementia and Amyotropic lateral sclerosis (right); and other complex brain disorders such as Epilepsy, Depression and brain tumor development (top). Relevant circRNAs (in red) have been shown to regulate the cell cycle of neural stem cells (radial glia -RG, intermediate progenitors–IP), the migration of newborn neurons (MN), the differentiation into inhibitory (IN) and excitatory (EN) post-mitotic neurons, astrocytes (A), microglia (MG), and oligodendrocytes (O), synaptogenesis, and mechanisms involved in mature brain function, homeostasis and survival- synaptic transmission and plasticity, neuronal and dendritic morphology, as well as brain immune activation.

Circular RNAs are a recently re-discovered, evolutionary conserved, large class of RNA molecules (Wang et al., 2014). Eukaryotic circRNAs are produced by a process called back-splicing, leading to the formation of a covalent bond between a pre-mRNA 3′-splice donor (down-stream) and a 5′-splice acceptor (up-stream) site, resulting in the generation of a unique junction, the back-splice sequence (BSJ) (Jeck et al., 2013; Memczak et al., 2013) (Figure 1A). Still, it is the canonical splicing machinery that generates these continuous loops of RNA while processing exons, introns, or both exon-introns of, primarily, protein coding genes. The precise details of circRNA biogenesis are not yet fully elucidated, but evidence underscores the importance of base-pairing of the two introns adjacent to the circularizing exons, with typically long length (>10 kilobases), enriched in inverted ALU repeats (Jeck et al., 2013). In other cases, specific binding of RBPs (such as FUS and QKI) to the intronic sequences adjacent to circularizing exons drives circularization (Ashwal-Fluss et al., 2014; Starke et al., 2015).

The circular conformation of these RNA molecules confers a unique resistance against degradation, since 3′–5′ exonucleases preferentially digest linear RNAs while leaving most circRNAs intact (Suzuki et al., 2006). Thus, circRNAs are much more stable than linear RNA transcripts (Hwang and Kim, 2024), with a median half-life that is at least 2.5 times longer (Enuka et al., 2016; Jeck et al., 2013). Nonetheless, most circRNAs are expressed at low levels when compared to their cognate linear counterparts (Santer et al., 2019), but in a highly tissue-, cell type-, and time point-dependent context. To date, based on large scale, bulk RNA sequencing (RNAseq) studies, multiple databases have been constructed to help annotate circRNA expression patterns of millions of circRNAs across different cell lines, tissues and organisms (Chen et al., 2016; Dong et al., 2018; Glažar et al., 2014; Meng et al., 2019; Wu et al., 2020). Furthermore, with the advent of single cell RNA sequencing, efforts have been made to resolve the complexity of circRNA expression landscapes also at the single cell level (Fan et al., 2015; W. Wu et al., 2022; Zhong et al., 2017).

While a molecular and functional characterization remains neglected for the majority of circRNAs, some circular RNA molecules that are primarily retained in the nucleus, have been described to act on the transcription and splicing regulation of their host and/or nearby located genes (Mai et al., 2022). Others are primarily exported into the cytoplasm, where they can act as miRNA “sponges” (Hansen et al., 2013; Peng et al., 2017), or interact with RBPs (Holdt et al., 2016; Ikeda et al., 2023), thus competing with linear mRNAs. Furthermore, some circRNAs appear to be translated in a cap-independent fashion, via internal ribosomal entry sites (IRES) (Chen et al., 2021), although the peptide coding potential of circRNAs, at endogenous levels and in physiological context, remains under discussion. CircRNAs expression is especially abundant in the brain and increases during aging, independently from the host gene mRNA expression (Hanan et al., 2017; Knupp and Miura, 2018; Rybak-Wolf et al., 2015). Over the last decade, abnormal levels of circRNAs have been associated with a large number of complex human neurologic disorders, ranging from ischemic stroke, over neurodevelopmental disorders (such as epilepsy, schizophrenia and autism spectrum disorders), Alzheimer’s Disease and Parkinson’s Disease, as well as Huntington’s Disease (Mai et al., 2022; Morandell et al., 2024; Titze-de-Almeida & Titze-de-Almeida, 2023; Wang et al., 2023).

Recent work has further identified the dedicated mechanism by which circRNAs are exported to the cytoplasm, dependent upon Ran-GTP/Exportin-2, underscoring the potential relevance of circRNA localization for cell function (Ngo et al., 2024).

While for a vast majority of the identified deregulated circRNAs associated with human neurologic disorders their biologic function and relevance for the disease pathogenesis/progression remains elusive, in the past decade a number of studies have discovered important regulatory roles for some circRNAs in neuronal development, function and homeostasis with the detailed analysis of their molecular mechanisms of action. This review will put these most recent functional studies into the spotlight and consequently discuss how deregulation of circRNAs can modify complex neurologic disorders, from neurodevelopment to neurodegeneration.

## CircRNAs in physiologic brain development and function

CircRNAs are most abundantly expressed in brain tissues (Hanan et al., 2017). The majority of neuronal circRNAs were shown to be localized both at the cell body and dendrites of neurons. Additionally, their expression pattern changes according to the developmental stage of the cell, suggesting a functional role of circRNAs during development and the mature brain (Hollensen et al., 2020; Venø et al., 2015). In line with these observations, circRNAs found in dopaminergic and pyramidal neurons are predominantly expressed from gene loci relevant for synaptic activity, underscoring their potential functional role in determining activity of these neuronal subtypes, but also the whole neuronal network (Dong et al., 2023; You et al., 2015). Single cell RNAseq efforts have furthermore shown that circRNAs display specific expression patterns in different neuronal cell types, presumably contributing to cell diversity and identity (Dong et al., 2023).

Arguably the most thoroughly studied neuronal circRNA is complementary determining region 1 antisense (*Cdr1-as*). *Cdr1-as* contains >70 binding sites for the neuronal microRNA (miRNA) *miR-7* and acts as *miR-7* sponge (Hansen et al., 2013), as part of a complex non-coding regulatory network (Kleaveland et al., 2018; Piwecka et al., 2017). Interestingly, binding of *miR-7* seems to actively mediate the circularization of *Cdr1-as* (Belter et al., 2022), while loss of *Cdr1-as* leads to miR-7 destabilization and alters excitatory synaptic transmission, driving schizophrenia-associated behaviours (Piwecka et al., 2017). Moreover, synaptic localization of *Cdr1-as* is required for fear-memory extinction (Zajaczkowski et al., 2023), while, in cortical neurons, *Cdr1-as* buffers *miR-7* activity to control glutamatergic excitatory transmission and neuronal connectivity. As such, these observations highlight the possible relevance of circRNAs for memory formation and long-lasting synaptic adaptations (Cerda-Jara et al., 2024).

Human *circSLC45A4*, one of the highest expressed circRNAs in the developing human frontal cortex, was reported to be necessary in maintaining the pool of neural progenitors *in vivo*, and its knock-down promoted Cajal-Retius cell differentiation in the cortical plate during early embryonic development (Suenkel et al., 2020). Similarly important for neural progenitor differentiation and cell positioning in the developing cortex is *circFAT3.* As shown by Seeler et al., it is especially relevant for differentiation of human embryonic stem cells into rostral and caudal neural progenitors. While *circFAT3* knock-down caused minimal changes in bulk RNA expression, it had significant effects at the single-cell level in human cerebral organoids, disrupting expression patterns linked to neural differentiation and migration of telencephalic radial glial cells and mature cortical neurons (Seeler et al., 2023).

Morphology and migration of midbrain dopamine neurons (mDA), crucial for motor and cognitive behaviours and linked to various brain disorders, are critically regulated by *circRmst* (Rybiczka-Tešulov et al., 2024). While single-molecule fluorescence *in situ* hybridization (smFISH) did not reveal any specific compartmentalization of highly expressed circRNAs in the cell, *in vitro* knock-down of the candidate *circRmst* had wide-spread effects in *in vitro* primary cultures, such as increased soma size, soma-to-nucleus ratio, reduced numbers of tyrosine hydroxylase positive neurons and, *in vivo*, accelerated neuronal migration and lateral mislocalization of the mDA neuron pool (Rybiczka-Tešulov et al., 2024). On the other hand, *circTulp4* depletion, also highly expressed in developing mDA neurons, only led to milder effects, causing a small decrease in neurite length and increase in secondary branch formation (Rybiczka-Tešulov et al., 2024). Its primary function, in fact, is thought to lie in the regulation of general brain physiology and synaptic activity, due to its compartmentalized enrichment at the synapse. Concordantly, depletion of *circTulp4* - by the alteration of the splice acceptor site responsible for its biogenesis - modulated the strength of excitatory neurotransmission in primary cultures and sensitivity to aversive stimuli in behavioral assays (Giusti et al., 2024). While *circTulp4* molecular mechanism is unlikely to be driven by miRNA-sponging or translation from the circular molecule, its mode of action remains to be identified (Giusti et al., 2024).

Synaptogenesis and dendritic spine formation was further shown to be strictly regulated by *circRERE and circPhf21a* expression in rat hippocampal neurons. *CircRERE* depletion led to the formation of electrophysiologically silent synapses, and Kelly et al. could demonstrate *circRERE*-mediated stabilization of *miR-128-3p* as a novel molecular mechanism to modulate the formation of silent excitatory synaptic co-clusters (Kelly et al., 2024, 2025).

The miRNA-sponging ability of circRNAs, implies also the capability of these molecules to regulate gene expression through circRNA/miRNA/mRNA interaction networks. In line with this molecular mechanism, *circDlc1* was shown to bind to *miR-130b-5p,* controlling its synaptic localization and regulating the expression of different mRNAs in the glutamatergic circuit. Thereby it plays an important role in synaptic transmission, regulating glutamatergic signaling and plasticity in cortico-striatal pathways. In line with these findings, loss of *circDlc1* lead to hyperactivity behaviour in a knock-out mouse model (Silenzi et al., 2024).

Taken together, these studies highlight important regulatory functions of circRNAs for physiologic brain development, modulating all steps of the process, from neural progenitor positioning and neurogenesis, over migration and differentiation of newborn neurons, to dendritic spine formation, synaptogenesis, synaptic function and plasticity (Figure 1B).

## CircRNAs in neurodevelopmental disorders

Because of their enrichment in the central nervous system (CNS) and their crucial roles in neurogenesis, synaptic plasticity and neuronal identity not surprisingly, circRNAs have been associated with neurodevelopmental (NDDs) and neuropsychiatric disorders, were they are increasingly recognized as key regulators of pathological processes (Nemeth et al., 2024). According to the DSM-5, NDDs include autism spectrum disorders (ASD), intellectual disability (ID), attention deficit hyperactivity disorder (ADHD), communication and motor disorders. These diagnostic categories, defined by specific sets of symptoms, show considerable comorbidity and phenotypic overlap, complicating diagnosis and management of the disorders. In fact, genetic research supports the hypothesis that ID, ASD, ADHD and schizophrenia lie on a neurodevelopmental continuum, rather than representing independent conditions (Morris-Rosendahl and Crocq, 2020).

The phenotypic heterogeneity of ASD and the co-morbid other neurodevelopmental syndromes is mirrored by the large complexity in underlying genetic architecture and altered biologic pathways, which have been studied in detail over the last 4 decades (reviewed here (Choi and An, 2021) and here (Parenti et al., 2020)). Instead, the exploration of long non-coding (lnc) RNAs, of which circRNAs are an important subgroup, and their contribution to the etiology of NDDs is yet in its infancy, and has been, so far, primarily focused on ASD and schizophrenia, but can possibly be extrapolated to other NDDs (Ghafouri-Fard et al., 2022; Jiang et al., 2023; Stott et al., 2023). A comprehensive study utilizing previously published RNAseq data sets from total RNA (ribosomal RNA-depleted RNAs without poly(A)-selection) derived from a large collection of *post mortem* brain tissue of control and ASD cases (Parikshak et al., 2016; Wu et al., 2016), represents the first systematic investigation of circRNA dysregulation in ASD. In the study, the authors identified 60 circRNAs and three co-regulated modules that were perturbed in ASD. By integrating circRNA, microRNA, and mRNA dysregulation data derived from the same cortical samples, 8170 ASD-associated circRNA/miRNA/mRNA networks were identified. The 2302 putative target genes of these axes were enriched for ASD risk genes and genes encoding inhibitory postsynaptic density (PSD) proteins, but not for genes implicated in monogenic forms of other brain disorders or genes encoding excitatory PSD proteins. Functionally, only *circARID1A* was tested: upregulated in ASD brain samples and working as *miR-204-3p* sponge, the corresponding circRNA/miRNA/mRNA regulatory network was further characterized *in vitro* employing knock-down and over-expression experiments, supporting the notion that circRNAs may serve as efficient miRNA sponges, influencing the expression dynamics of ASD-associated risk genes and leading to synaptic dysfunction and abnormal neural development (Huang et al., 2023). Furthermore, the genomic study by Te-Lun Mai et al. focused on quantitative trait loci (QTL) associated with the expression of nearby circRNAs (circQTL). They integrated RNAseq and genotyping data from *post mortem* brain tissues of individuals with ASD, to identify circQTLs that *cis*-regulate nearby circRNAs expression and, in turn, *trans*-regulate distant gene expression (*trans*-eGenes). The researchers identified 3,619 circQTLs, constructing 19,804 circQTL-circRNA-trans-eGene regulatory axes. 291 of these circQTLs overlapped with ASD-related genetic variants identified in genome-wide association studies (GWAS), suggesting a potential link between these circQTLs, SFARI risk factor genes, and ASD pathogenesis (Mai et al., 2022). Such findings highlight the complexity of gene regulation in ASD and suggest that circRNAs may serve as key intermediaries in the genetic architecture of the disorder.

On the other hand, additional insights on the importance of circRNAs in NDDs can be derived from studies using animal models. Wang et al. identified a vast array of differentially expressed circRNAs following valproate treatment in the mouse brain. By exploring a circRNA-based competing endogenous RNA (ceRNA) network, the study revealed possible interactions between circRNAs, miRNAs, and mRNAs, highlighting the complex regulatory networks that might potentially contribute to neurodevelopmental pathology (Wang et al., 2021). Similarly, Gasparini et al. explored the differential expression of hippocampal circRNAs, such as *circCdh9*, in the BTBR mouse model of ASD (Meyza and Blanchard, 2017). Their findings suggest that these circRNAs may contribute to synaptic dysfunctions observed in ASD (Gasparini et al., 2020).

In the context of SCZ, *circHOMER1* expression levels were significantly reduced in *post mortem* orbitofrontal cortex samples of patients, and *circHOMER1* levels positively correlated with the age of SCZ onset. *CircHomer1a* regulated the expression of specific isoforms of synaptic plasticity-related genes with relevance for psychiatric disorders. Knock-down of *circHomer1a* in the orbitofrontal cortex was sufficient to disrupt cognitive flexibility *in vivo* (Zimmerman et al., 2020). In SCZ, circRNAs have also been intensely studied as potential class of novel diagnostic and therapeutic biomarkers (Huang et al., 2023; Tan et al., 2021; Yao et al., 2019). For instance, circRNA expression was profiled in plasma samples of very early-onset schizophrenia (VEOS) patients and 235 circRNAs (1 upregulated, 234 downregulated) were shown to be differentially expressed. A circRNA/miRNA/mRNA network was then constructed and identified target genes that were involved in signal transduction, the cytoskeleton and transport pathways. Thus, plasma circRNAs in this network may be potential diagnostic biomarkers for VEOS and could play an important role in the onset and development of VEOS symptoms (Huang et al., 2023).

Taken together, although with much room for further exploration, circRNA dysregulation might lead to aberrant gene expression, contributing to the development and progression of neurodevelopmental and neuropsychiatric disorders. As modulators of altered neurodevelopment, they may also represent promising therapeutic targets, offering new avenues for treating neuropsychiatric conditions or be used as diagnostic or therapeutic biomarkers in easily accessible biofluids.

## CircRNAs in the aging brain and neurodegenerative diseases

Neurodegenerative disorders, including Alzheimer’s disease (AD), Parkinson’s disease (PD), amyotrophic lateral sclerosis (ALS), and Huntington’s disease (HD), are marked by the progressive degeneration and loss of neurons in distinct regions of the brain and spinal cord. These conditions are currently irreversible, with no effective treatments available, eventually leading to decline and death of the affected patient. Although, in certain cases, clear genetic causes can be identified, aging still represents an important risk factor for most neurodegenerative diseases (Hou et al., 2019). Thus, in line with the ever increasing life expectancy and increasing numbers of individuals with advanced age, especially in industrialized countries, neurodegenerative disorders are considered a leading cause of death by the World Health Organization (WHO).

### Alzheimer’s disease

Global changes in circRNA expression levels in AD have first been explored in detail by Dube et al., demonstrating that collectively, the expression changes of ten circRNAs, in the human *post mortem* parietal cortex, had a higher predictive value for cognitive impairment and dementia, than, e.g., the number of *APOE4* alleles (Dube et al., 2019). Additionally, an association of circRNA expression levels with AD has also been found in other brain regions (Cochran et al., 2021; Lo et al., 2020), such as the frontal lobe, temporal cortex and hippocampus. Importantly, circRNAs from two AD-associated genes, *presenilin 1* (*PSEN1*) and *tau* have further been studied more in detail. *CircPSEN1* levels increase during autosomal-dominant Alzheimer’s disease progression which may be related to neuro-inflammatory events and amyloid beta accumulation (Chen et al., 2022). Instead, after undergoing RNA editing, circular RNAs from the *tau* pre-mRNA encompassing the microtubule binding sites were shown to be translated into proteins that promote tau aggregation *in vitro,* and are therefore hypothesized to contribute to tauopathies more in general (Welden et al., 2022).

Maquera et al. provided initial evidence for a role of translated circRNAs in the development of AD. Through RNAseq based analysis of the mRNA and circRNA composition in *post mortem* human cortical tissues during AD progression, as characterized by the spread of neurofibrillary tangles from the entorhinal cortex (early stages) to the whole brain, they identified *circMAN2A1* expression to be correlated with AD progression. Transfection experiments indicated that RNA editing promoted its translation using start codons out of frame with linear mRNAs, which generates novel proteins (Arizaca Maquera et al., 2023).

In a study by Puri et al., RNAseq analysis of neuropathologically confirmed AD brains and controls (n = 2027) identified 4,092 deregulated circRNAs in the hippocampus and 4,912 in the cortex. The study aimed to assess the relationship between circRNA expression patterns and AD clinical and pathological outcomes (Puri et al., 2023). CircRNA expression was correlated with the Clinical Dementia Rating (CDR) and AD-related neuropathological traits, including CERAD neuritic plaque score and Braak stage. Among 48 differentially expressed circRNAs, 69% were significantly associated with all three parameters. Key circRNAs such as *circSLC8A1* (linked also with Parkinson’s disease) and *circHOMER1*, associated with synaptic function and calcium signaling, were significantly downregulated in AD patients, correlating with synaptic loss and cognitive decline. The downregulation of the validated circRNAs following treatment with oligomeric Tau (oTau) highlighted the responsiveness of AD-associated circRNAs in neuro progenitor cells (NPC). *CircMAPK9* exhibited downregulation by oTau exposure, independent of its linear transcript counterpart. These observations raised the possibility that some of the changes in circRNA expression in the AD brain might reflect the response to oTau toxicity, consistent with Cervera-Carles et al., who noted similar responses of circRNAs between FTD-tau (but not FTD-TDP-43) and AD (Cervera-Carles et al., 2020).

Using the 5xFAD genetic mouse model, which exhibits early Aβ plaque deposition and progressive cognitive decline (Forner et al., 2021), Wang et al., investigated circRNA alterations at critical disease stages (5 and 7 months). A-tailing RNAseq combined with the CARP algorithm revealed 342 and 467 differentially expressed (DE) circRNAs in the cortex at these time points, with additional 264 DE circRNAs in the hippocampus (Wang et al., 2024). To determine human relevance, they compared these findings with RNAseq data from the Mount Sinai Brain Bank (MSBB), confirming that many DE circRNAs were also dysregulated in AD patient brains. One of the most significantly altered circRNAs, *circGigyf2*, was highly expressed in healthy brains but showed marked downregulation at 7 months in 5xFAD mice and in human AD cortical regions. Notably, *circGigyf2* interacts with CPSF6, a critical RNA-processing factor involved in 3′-end cleavage and polyadenylation. Its loss correlated with increased CPSF6 availability, leading to altered poly-(A)-site (PAS) selection and disrupted gene expression in thousands of transcripts, suggesting a role in AD pathogenesis. They also observed alternative splicing events within circRNAs, affecting their interactions with miRNAs and RBPs. For example, *circCcdc50* exhibited exon 4 skipping in the 7-month 5xFAD cortex, impacting *miR-335-5p*, a miRNA linked to AD-related gene expression changes. More broadly, miRNA sequestration appeared to be dynamically regulated by circRNA clusters, where multiple low-abundance circRNAs collectively influenced miRNA activity. Although individual circRNAs within a cluster may not achieve statistical significance in DE analysis, they can collectively function as a regulatory unit.

Additionally, Li et al. found that *circAXL* plays a key role in regulating BACE1, a protein associated with amyloid-beta production (Li et al., 2022). Over-expression of *circAXL*, which, in turn, negatively regulated *miR-328*, led to increased neuronal apoptosis, reduced neurite outgrowth, and heightened inflammation in AD models. In contrast, *miR-328* over-expression had protective effects, suggesting that *circAXL* and its modulation of BACE1 through *miR-328* may serve as potential therapeutic targets for AD.

These findings demonstrate that circRNAs are not passive byproducts of splicing but active regulators of gene expression in AD, as they affect both miRNA function and RNA-processing pathways. Their brain region-specific disruptions observed in both mice and AD patients highlight their potential as biomarkers and therapeutic targets in neurodegeneration.

### Parkinson’s disease

Secondly to AD, PD is one of the most common neurodegenerative disorders with loss of dopamine production from substantia nigra dopaminergic neurons, leading to overt motor impairment and psychological decline. RNAseq studies on PD mouse models highlighted several dysregulated circRNAs, among which *circSV2b* which was further studied. Evidence shows that its over-expression, in PD induced mice, results in the restoration of dopamine synthesis and a normal nigro-striatal function. *CircSV2b* regulates the activity of *mirR-5107-5p* which modulates the transcription of *Foxk1* mRNA and acts on the *Akt1* signaling pathway, participating in PD–related oxidation. These data suggest that *circSV2b* may not only be investigated to understand the pathogenesis of PD, but could also be a promising candidate for PD treatment or even exploited as a molecular biomarker (Cheng et al., 2022). Furthermore, studies on PD patients revealed an over-expression of *circSLC8A1,* predicted to bind *mir-128,* in patients. Over-expression of this circRNA is strongly linked to the oxidative stress, a crucial characteristic of PD and most neurodegenerative disorders (Hanan et al., 2020).

### Frontotemporal dementia

The human microtubule-associated protein tau (MAPT) stabilizes microtubules in neurons through up to four microtubule binding repeats, mainly formed by exons 7–12. Exon 10 - encoding the second microtubule-binding repeat - is alternatively spliced in the adult human brain leading to a mixture of tau proteins with 3 or 4 microtubule binding repeats. This ratio can be altered in disease, especially in several forms of frontotemporal dementia with parkinsonism linked to chromosome 17 (*FTDP-17*) (Corsi et al., 2022). Margvelani et al. investigated the impact of *FTDP-17* mutations on the formation and translation of circular RNAs derived from the *MAPT* gene (Margvelani et al., 2023). Interestingly, five *FTDP-17* mutations increased the formation of circRNA involving back-splicing from exon 12 to exon 7 (*circTau (7,8,9,10,11,12)*), while 3 other different mutations elevated the levels of circRNA from exon 12 to exon 10 (*circTau(10,11,12)*). These circRNAs undergo RNA editing through the interferon-induced ADAR1-p150 isoform which promotes the translation of *circTau* RNAs.

Atrian et al. further support the role of circRNAs and N6-methyladenosine (m6A) modification, a key epitranscriptome mark affecting RNA stability and processing, in tau-induced neurotoxicity, by using both *Drosophila* models and human induced pluripotent stem cell (iPSC)-derived neurons. RNAseq analysis found, among others, *circMbl* to be significantly upregulated in tauopathy models. Depletion of *circMbl* reduced tau-induced cell death in *Drosophila* neurons, suggesting that increased *circMbl* contributes to neurotoxicity. Further experiments revealed an overall increase in m6A levels, which led to enhanced back-splicing and circRNA biogenesis in *tau* transgenic *Drosophila*. By m6A-RIP sequencing, tau-induced *circMbl* upregulation was confirmed to be m6A-dependent with suppression of RNA methylation factors mitigating tau-induced neurotoxicity. Taken together, these findings underscore a complex interplay between RNA editing and *FTDP-17* mutations in regulating circRNA dynamics and their potential contribution to disease pathology. On the other hand, they also highlight a novel molecular mechanism where tau-driven m6A modifications might promote circRNA formation and contribute to neurotoxicity, thus opening a possible therapeutic strategy for tauopathies (Atrian et al., 2024).

### Amyotrophic lateral sclerosis

Amyotrophic lateral sclerosis (ALS) is a progressive neurodegenerative disease affecting both upper and lower motor neurons, leading to muscle atrophy, speech difficulties, and respiratory failure (Brown and Al-Chalabi, 2017). With an average life expectancy of just 30 months from symptom onset, early and accurate diagnosis is critical (Hardiman et al., 2017). Given the variability in clinical presentation, especially in patients without genetic mutations or familial history, reliable biomarkers are essential for timely detection and intervention. There are very few studies available exploring the role of circRNAs in ALS, with most focusing on their dysregulation rather than their functional significance. For instance, Dolinar et al. identified 274 upregulated and 151 downregulated circRNAs in ALS patients compared to healthy controls using microarray assays. Based on these findings and the functional relevance of their host genes in ALS, 10 circRNAs were selected for further PCR validation in a larger sample set. Of the ones experimentally validated, two are of particular interest based on their host gene identity, namely *hsa_circ_0000567*, which is derived from *SETD3*, linked to muscle differentiation, and *hsa_circ_0023919*, derived from *PICALM*, which plays a role in clathrin-mediated endocytosis at neuromuscular junctions and has been associated with AD. Notably, all selected circRNAs contain predicted binding sites for one or more RBPs. Further analysis revealed that *hsa_circ_0000567*, *hsa_circ_0023919*, and *hsa_circ_0088036* were negatively correlated with patient age at blood collection, while *hsa_circ_0000567* and *hsa_circ_0088036* were also negatively associated with the age of disease onset (Dolinar et al., 2019).

Aquilina-Reid et al. assessed circRNA expression levels in spinal cord tissues and explored the functional interactions between circRNAs, miRNAs and mRNAs in ALS, identifying 92 differentially expressed circRNAs (Aquilina-Reid et al., 2022). TargetScanHuman database based prediction of targeted mRNAs by selected miRNAs, then, revealed 55 genes whose targets were both significantly upregulated and differentially expressed in ALS (Friedman et al., 2009; Garcia et al., 2011; Grimson et al., 2007; Lewis et al., 2005). These genes were enriched for Gene Ontology terms such as “cell-cell signaling by Wnt” and “positive regulation of proteasomal ubiquitin-dependent protein catabolic process,” both linked to ALS pathology (Chen et al., 2012).

Taken together these findings first demonstrate the potential significance of circRNA in ALS progression and for biomarker development, although follow up experiments are needed to validate these initial observations.

### Huntington’s disease

Huntington’s disease (HD) is a hereditary, fatal neurodegenerative disorder caused by a CAG trinucleotide expansion within exon 1 of the *HTT* gene. A polymorphic CAG tract up to 35 repeats is found in unaffected individuals, whereas alleles bearing 39 or more repeats lead to HD symptoms. Among the various dysfunctional mechanisms linked to the disease, RNA processing and alternative splicing (AS) alterations are emerging as crucial regulators of HD (Nguyen et al., 2025). Coherently, as the splicing machinery is also instrumental to circRNA biogenesis, different evidence is suggesting a functional role of circRNAs in HD. An initial study, using a circRNA microarray, compared expression patterns of rat cell lines expressing mutant or wild-type huntingtin (Marfil-Marin et al., 2021). A total of 23 circRNAs were differentially expressed, most of which downregulated. CircRNA/miRNA/mRNAs interactions were predicted, suggesting that changes in these regulatory networks might impact neuronal function and survival in HD. Capitalizing on accurate genetic mouse models of the disease, Ayyildiz et al. examined AS and circRNA biogenesis in HD by RNAseq approach (Ayyildiz et al., 2023). While circRNA expression was generally higher in NPCs compared to embryonic stem cells (ESC), cells expressing mutant huntingtin exhibit a mis-shaped repertoire of linear and back-spliced circular RNA isoforms. A negative correlation between CAG length and circRNA expression was noted in NPCs, suggesting a mutation-dependent disruption of circularization. A significant proportion (32.7%) of differentially expressed circRNAs were found to be modified by m6A. This indicates that mutant huntingtin may impact circRNA biogenesis through m6A-mediated regulation. Interestingly, these splicing defects were most prominent in neural progenitors, with minimal changes observed in ESCs, highlighting the early vulnerability of neural cells to RNA dysregulation in HD. Interestingly, a highly conserved circRNA is also stemming from the HD gene locus itself, *circHTT(2,3,4,5,6)*. *CircHTT(2,3,4,5,6)* expression significantly correlates with the length of the CAG-repeat tract in exon-1 of *HTT* in human and mouse HD model systems. Mouse *circHtt(2,3,4,5,6)*, is expressed during embryogenesis and increases during nervous system development. *CircHtt(2,3,4,5,6)* over-expression experiments in the HD-relevant ST*Hdh* striatal cells further revealed its ability to modulate CAG expansion-driven cellular defects in cell-to-substrate adhesion, acting as novel modifier of HD pathology and potential drug target (Morandell et al., 2024). These three studies underscore the potential for targeting splicing, back-splicing and RNA modifications as therapeutic strategies in HD.

## CircRNAs in other disorders of the CNS

Deregulation of circRNAs has also been described in the context of other disorders of the central nervous system, that are not strictly of neurodevelopmental or neurodegenerative nature. These disorders range from epilepsy, depression and bipolar disorder, the inflammatory disease multiple sclerosis, to brain tumors. All these conditions are similarly complex and heterogeneous in their etiology, phenotypic composition and therapeutic management, as the typical neurodevelopmental and neurodegenerative disorders.

### Temporal lobe epilepsy

CircRNAs can regulate synaptogenesis and synaptic transmission, thus they are directly linked with neural network activity and the excitation-inhibition interplay in the brain. In line with this notion, numerous circRNAs have been investigated to provide a better understanding of the pathogenesis of mesial temporal lobe epilepsy (mTLE), the most common form of refractory focal epilepsy. Through high-throughput sequencing of mTLE patients-derived samples, thousands of circRNAs were identified; *circSATB1*, *circSATB2* and *cirLRP6* showed an interesting downregulation in the hippocampus of patients when compared to controls. Functionally, *circSatb1* regulates dendritic spine morphology and function (Venø et al., 2015). The deregulated expression of circRNAs in mTLE was also confirmed by Gomes-Duarte et al., highlighting aberrant increase in *circArhgap4* and *circNav3* expression from early to late stage of experimental epilepsy, likely as consequence of recurrent spontaneous seizures. Abnormal electrical brain activity, in turn, could be potentially correlated to the bioavailability of miRNA pathophysiologic drivers, binding targets of the dysregulated circRNAs with predicted miRNA-sponging activity (Gomes-Duarte et al., 2021). The hypothesis that circRNAs may interfere with the abundance and availability of miRNAs in the context of TLE is particularly relevant since different studies previously reported dysregulated miRNA levels in TLE patients’ circulation, miRNAs as disease modifiers as well as their importance for drug resistance in TLE [reviewed here (Guarnieri et al., 2024)].

### Depression

Major depressive disorder (MDD) is a very common disorder, severely limiting psychosocial functioning and diminishing quality of life of affected individuals. The WHO ranked MDD as the third cause of burden of disease worldwide and projected that it will rank first by 2030 (Malhi and Mann, 2018). The etiology of MDD is believed to be multifactorial, with biological, genetic, environmental, and psychosocial contributing factors (Cui et al., 2024). Pathophysiologic mechanisms include neuroinflammation and alterations in neuroplasticity and epigenetics. Several long noncoding RNAs, including miRNAs and circRNAs, have been found to be deregulated in MDD, and could represent potential biomarkers for clinical diagnosis and monitoring of the therapeutic outcomes (Shi et al., 2021b). A small pilot study on 129 participants showed that individuals affected by MDD have increased plasma levels of *circHIPK2* (Yu et al., 2024), which is consistent with previous findings in a mouse model system of depression (Zhang et al., 2019). In this study, the authors also speculated about a possible involvement of *circHIPK2* in the gut-brain axis*:* Previously implicated in astrocyte activation in brain physiology (Huang et al., 2017), alterations of *circHIPK2*-regulated astrocyte activity in response to changes in the microbiome could modify the MDD behavioral characteristics (Hao et al., 2022; Zhang et al., 2019).

Furthermore, data from samples of MDD patients and two mouse models of depression (chronic unpredictable stress (CUS) and lipopolysaccharide (LPS) models), revealed increased *circMBML1* levels and reduced expression of *circFKBP8* (Shi et al., 2021a)*, hsa_circRNA_103636* (Cui et al., 2016) and *circDYM(4,5,6)* (Zhang et al., 2020) in peripheral blood samples. Interestingly, overexpression of *circDym(4,5,6)* in the mouse hippocampus, regulated immune activation by sponging miR-9, which, in turn, led to a consequent decrease of microglial activation upon CUS and LPS treatment, a hallmark of MDD, with depression-like behaviors ameliorated in these mice (Zhang et al., 2020). These findings suggest that, i) peripheral blood *circDYM(4,5,6)* levels may represent a promising biomarker, and ii) *circDYM(4,5,6)* over-expression may represent a novel treatment strategy for MDD. While these represent exciting initial observations, follow up studies in independent patient cohorts, alternative mouse models of depression and human *in vitro* model systems are imperative to assess the true value of these findings for clinical translation.

### Bipolar disorder

Bipolar disorder (BD) is a psychiatric disorder characterized by the alternation of depressive and maniacal phases during the lifetime of the affected individuals (Tondo et al., 2017). In recent years, studies explored the role of gene regulation and non-coding RNAs in the pathophysiology of BD. Analyzing the expression of circRNAs in peripheral blood mononuclear cells (PBMCs) from patients with BD, in comparison to samples from a non-psychiatric group, found around 33 circRNAs to be significantly dysregulated (Mahmoudi et al., 2021). Additionally, RNAseq analysis on samples from anterior cingulate cortex of individuals with BD compared to neurotypical controls, identified several dysregulated circRNAs among which, *circCCNT2* showed a significant upregulation in the BD cohort (Lin et al., 2021). Many studies focused on the function of *circHomer1* as a psychiatric disease-related circular RNA: its expression is reduced in BD as well as other diseases such as SCZ. Using knock-down mouse models, low levels of *circHomer1* were shown to be associated with changes in animal cognitive ability and behaviour, possibly explained by *circHomer1* ability to modulate the expression of genes involved in synaptic plasticity, thus affecting neuronal function (Zimmerman et al., 2020, 2024).

The differential expression of *circNEBL* and *circEPHA3* - altered in RNAseq data from *post-mortem* brains of BD patients versus healthy controls - could further provide new insights into the role of circRNAs in BD (Luykx et al., 2019). Specifically, the upregulation of *circEPHA3* may be of particular relevance since EPHA3’s role in mediating axon growth (Javier-Torrent et al., 2019) could be linked to the neurodevelopmental aspects of BD.

### Multiple sclerosis

Multiple sclerosis (MS) is a complex autoimmune disease of the CNS characterized by demyelination, chronic inflammation, neuronal loss, and axonal damage (Thompson et al., 2018). The most common form, affecting 80% of patients, is relapsing-remitting MS, characterized by attacks followed by complete or partial remissions (Lublin et al., 2014). Although the pathogenic factors underlying MS remain largely unknown, several recent studies pointed to alterations in AS and RNA processing as new molecular mechanisms potentially involved in the pathogenesis. Coherently, some AS events as well as ncRNAs have been proposed as novel biomarkers for MS (D’Ambrosio et al., 2015; Hecker et al., 2019; Paraboschi et al., 2015; Spurlock et al., 2015). While functional investigation of circRNAs has been largely neglected, circRNAs were first implicated in MS by Cardamone et al., identifying the *GSMDB* derived *hsa_circ_0106803* circRNA as upregulated in PBMCs of 30 RR-MS patients compared to 30 healthy controls (Cardamone et al., 2017). Subsequently, Iparraguirre and colleagues detected 406 differentially expressed circRNAs through a microarray analysis on peripheral blood leucocytes of four RR-MS patients and four healthy controls; two downregulated circRNAs, both deriving from the *ANXA2* host gene, were validated in an independent cohort of 20 RR-MS patients and 18 healthy controls (Iparraguirre et al., 2017). In addition, through an RNAseq based analysis of blood samples, a global, specific and sex-dependent deregulation of circRNA expression in MS was observed in a cohort of 30 patients and 20 controls, with candidate circRNAs further examined in a second, independent cohort of 70 M cases, revealing 6 upregulated circRNAs (e.g., *circPADI4*) as potential biomarkers (Iparraguirre et al., 2020). CircRNAs were furthermore shown to be enriched at MS *loci*, emerging from genome wide studies, with further characterization of *hsa_circ_0043813* derived from the MS-associated *STAT3* gene (Paraboschi et al., 2018). Similarly, expression analysis in another patient cohort, revealed 166 differentially expressed circRNAs in MS patients, 125 of which were downregulated. One of the top dysregulated circRNAs, *hsa_circ_0007990*, derives from the *PGAP3* gene, relevant for the control of autoimmune responses. Later analysis of two independent replication cohorts confirmed the downregulation of *circPGAP3* suggesting its implementation as a possible RNA-based biomarker (Cardamone et al., 2023).

### Brain tumor development

CircRNAs play a complex role in cancer development, influencing tumor progression through exosomal transfer and facilitating communication between cancer cells and the surrounding microenvironment. Certain circRNAs can contribute to metastasis by promoting pre-metastatic niches (Dai et al., 2018; Wang et al., 2019). Additionally, the failure of transcription termination and uncontrolled gene transcription into the downstream gene followed by back-splicing can give rise to read-through circRNAs, which incorporate exons from adjacent genes (Liang et al., 2017; Vo et al., 2019). These aberrant circRNAs are associated with oncogenic processes, potentially driving tumor growth and malignant transformation.

Invasion and metastasis of tumor cells are the primary causes of mortality in patients with malignant tumors (Asif et al., 2021). Despite the growing interest in circRNAs in cancer, few studies specifically address their role in brain tumor development (Ahmed et al., 2022; Katsushima et al., 2023; Zhang et al., 2024). Among the multiple pathways implicated in circRNA-mediated tumorigenesis, such as p53, JAK/STAT, and ERK signaling, the PI3K/AKT/mTOR axis is particularly significant.

mTOR is a key downstream signaling molecule in the AMPK, MAPK, and PI3K/AKT pathways, playing a central role in cancer progression (Tee et al., 2003). PI3K activation promotes the transition from PIP2 to PIP3, a process reversed by PTEN, and subsequently leads to mTORC1 and mTORC2 activation, either directly or through Rheb regulation via TSC1/2 inhibition (Ma et al., 2005).

Xin et al. (2019) identified *hsa_circ_0067934* as a critical modulator of glioblastoma progression through its regulation of the epithelial-mesenchymal transition (EMT) process and the PI3K/AKT/mTOR signaling pathway. In their study, they analyzed 157 human brain samples from glioblastoma patients and healthy controls. qRT-PCR revealed that *hsa_circ_0067934* was significantly overexpressed in glioblastoma tissues compared to adjacent noncancerous tissues. To investigate the functional role of *hsa_circ_0067934*, siRNA-mediated knock-down experiments were conducted *in vitro*. The results demonstrated a significant reduction in cell proliferation of glioblastoma cell lines post-transfection, while apoptosis levels increased notably upon *hsa_circ_0067934* silencing. *Hsa_circ_0067934* knock-down, furthermore, significantly impaired the migratory ability of glioblastoma cells in wound healing assays. At molecular level, *hsa_circ_0067934* silencing resulted in a marked decrease of phosphorylated PI3K and phosphorylated AKT, while total PI3K and AKT levels remained unchanged. This suggests that *hsa_circ_0067934* promotes glioblastoma progression by activating the PI3K-AKT signaling pathway (Xin et al., 2019).

Moreover, Bian et al., 2018 identified *circ-CFH* as a novel oncogenic circRNA, upregulated in glioma tissues. *Hsa_circ_0015758* (*circ-CFH*) is a single exon-derived circRNA transcribed from the GRCh37/hg19 fragment of chromosome 1, sharing homology with the protein-coding gene complement factor H (*CFH*) and functioning as a *miR-149* sponge, thereby upregulating *AKT1* expression. To investigate the biological role of *circ-CFH* in glioma cells, siRNA-mediated knock-down was performed. Silencing *circ-CFH* significantly decreased cell proliferation and cells’ ability to form colonies *in vitro*, while subcutaneous injection of *circ-CFH* -suppressing cells into nude mice significantly decreased tumor volume and weight. Finally, the RNA sponging activity of *circ-CFH* was explored, confirming miR-149 as a direct target through luciferase reporter assays, regulating miR-149-target mRNAs, such as *AKT1* mRNA (Bian et al., 2018). Taken together, these studies highlight the possibility for circRNAs targeting in order to modulate the activity of the PI3K/AKTAkt/mTOR signaling pathway in the context of brain tumor development.

## CircRNAs as biomarkers for brain disorders

Circularity renders RNA largely resistant to exonuclease cleavage and, therefore, circRNAs are extremely stable, as demonstrated by their long half lives in cells (Memczak et al., 2015). Furthermore, they show remarkable tissue specificity in their expression patterns, can be secreted into the extracellular space via extracellular vesicles, - both exosomes (30–100 nm) and microvesicles (100–1,000 nm) (Hon et al., 2019; Lasda and Parker, 2016; Zhao et al., 2024), and have previously been detected in easily accessible biofluids such as saliva, blood and urine (Hutchins et al., 2021; Memczak et al., 2015; Zhao et al., 2018). All these characteristics render circRNAs ideal candidates for being reliable biomarkers. Biomarkers can support diagnosis, provide information about disease prognosis, and progression, and can be used to monitor the efficacy and safety of drug therapy. In fact, when it comes to complex brain disorders, reliable peripheral biomarkers would be extremely useful. Disease relevant circRNAs could enter the blood via blood brain barrier leakage and be measured in biofluids. They could aid 1) diagnosis, which, for instance, is still very difficult in ASD and ALS (Hus and Segal, 2021), 2) disease progression and staging, which is critical for clinical trial design and endpoint assessment, 3) patient care, as sampling is much less invasive when compared with the collection of cerebrospinal fluid, as commonly done in HD (Pellegrini et al., 2022). In line with these requirements, recently, a study utilizing two large publicly available datasets with longitudinal clinical and blood transcriptomic data of PD patients, identified 71 circRNAs that were sufficient to differentiate between genetic PD, at risk-individuals and controls, highlighting the potential of these circRNAs to aid PD diagnosis. Importantly, five circRNAs significantly correlated with PD severity, yet the identified circRNAs had no better predictive value than linear RNA, limiting their utility (Beric et al., 2024). This example, amongst many others discussed above, highlights important considerations in using circRNAs as biomarkers for complex brain disorders. First, most studies that are available to date and propose individual circRNAs as biomarkers are based on small sample sizes, i.e., few patients, and limited datasets, and only few have validated their primary findings in additional, independent patient cohorts. Secondly, gender-differences have not been taken into consideration in the majority of cases, which could hamper their potential for clinical translation. And third, the tissue-origin of the deregulated circRNAs proposed as biomarkers remains unexplored, thus it is unclear whether they truly represent a “window into the diseased brain” or whether their deregulation is a secondary, unspecific result. Therefore, more standardised and carefully designed studies, will clarify their true potential as biomarkers in the future and for other neurologic disorders.

### Moving forward: challenges and outstanding questions

**FIGURE 2:**
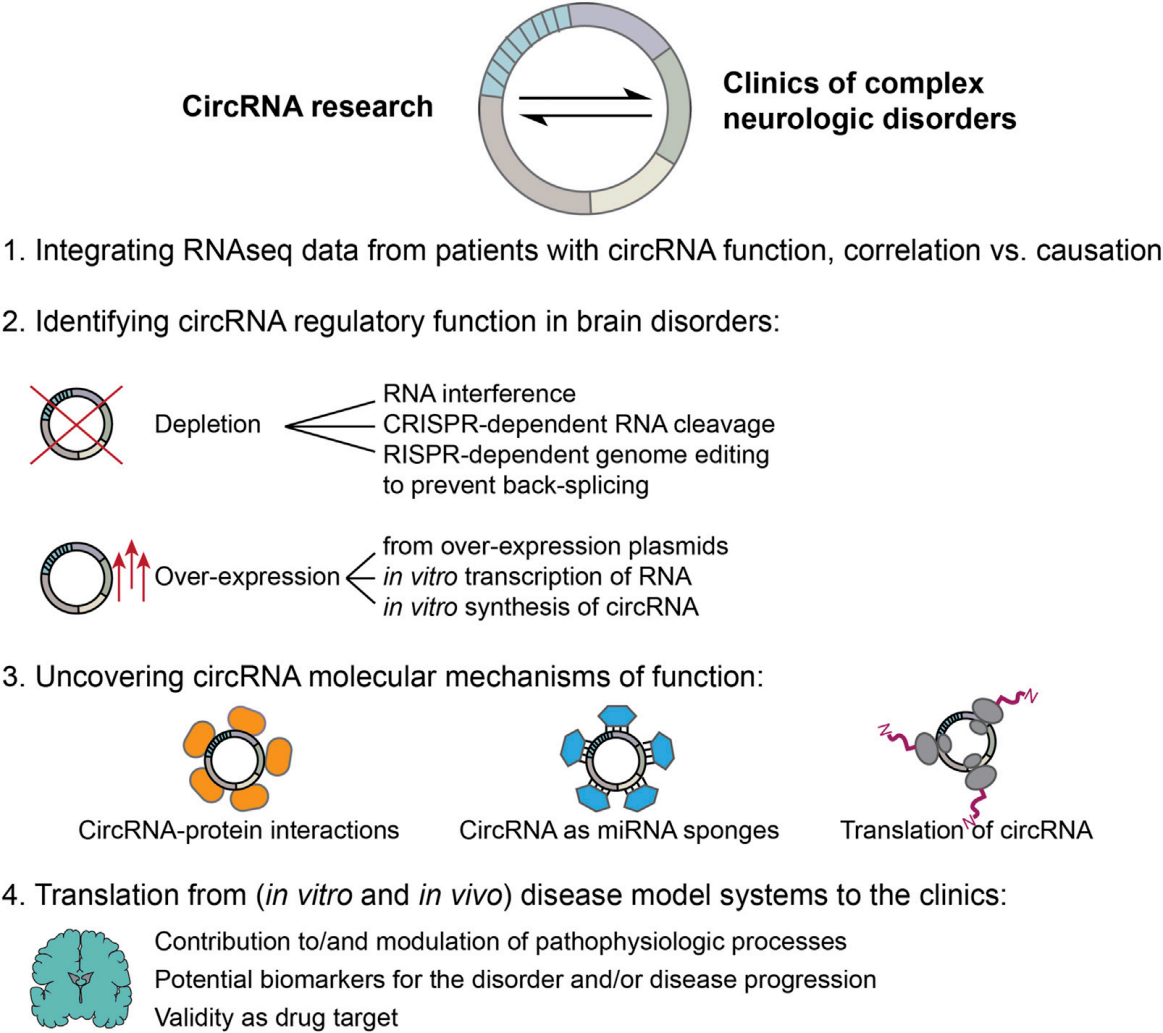
Moving forward: steps towards integrating results from circRNA basic research with the clinics of complex neurologic disorders. 1. Numerous RNA sequencing studies have found deregulated circRNA expression levels in samples derived from human patients. However, much fewer studies were able to experimentally validate a functional role and its molecular mechanism for the identified circRNAs in the pathophysiologic processes of the respective disorder. Future studies need to address this current gap in knowledge to avoid fallacy of causation. 2. This requires high quality follow up-experiments to identify circRNA regulatory functions. Experimental approaches can be based on circRNA depletion as well as over-expression. Both strategies face methodological difficulties unique to the circRNA field. CircRNA depletion approaches need to be highly specific to exclusively target the circRNA without simultaneously changing the linear RNA counterparts, or other unintended off-targets. Targeting circRNAs is especially difficult as sequence specificity is restricted to the back-splice junction, while genomic removal of sites required for back-splicing may unintentionally also alter linear splicing dynamics. Over-expression approaches, instead, can lead to difficult interpretation, since linear cognate RNAs can be produced (from plasmids), or co-transfected with *in vitro* transcribed or synthetized circRNAs; unintended immune activation may be induced, while over-expressed circRNA levels may reach extremely high levels leading to cellular effects that could be irrelevant in the physiologic context. 3. If depletion or over-expression of a circRNA of interest produces an effect, the underlying molecular mechanism should be investigated. A number of different molecular mechanisms have been proposed for circRNAs, including sequestration of RNA-binding proteins or activity as protein scaffolds, sponging of miRNAs as well as the production of circRNA related peptides through translation. When molecular mechanisms of function are investigated, it is of utmost importance to first determine endogenous expression levels of the involved components in the disorder of interest, to be able to draw physiologically relevant conclusions. 4. Finally, findings obtained from studying *in vivo* and *in vitro* model systems need to be translated back to the clinics in order to be useful for patients suffering from complex neurologic disorders. To that end, evaluation of a true contribution of the circRNA molecular function to the pathophysiologic processes, either on initiation or progression of the disease or modulation thereof, and/or its potential as biomarker as well as novel drug target need to be carefully assessed and tested.

The last decade has seen the circRNA research field explode, and experimental evidence highlighting the important regulatory functions of these peculiar molecules for brain physiology and complex neurologic disorders has continuously piled up over the years. Nonetheless, despite the rightful excitement about the discovery of a new regulatory layer that could modulate still untreatable, severe and -in some cases- lethal disorders, measures need to be taken to validate findings derived from RNAseq studies of *post mortem* human, or model organism brain samples, demonstrating a clear functional role of the deregulated circRNAs in the pathogenesis, and/or disease progression by experimental scrutiny to avoid misinterpreting correlation with causation. The circRNA field thereby faces unique challenges, caused by the -mostly- low expression levels, extremely high sequence similarity with the linear RNA counterparts, as well as high stability of these molecules, in addition to issues related to a lack of standardized protocols and detection tools that limit the precision and specificity of experimental approaches. In Figure 2 we suggest a roadmap in moving the circRNA field from performing RNAseq studies, to being able to eventually translate discoveries back to the clinics in order to benefit patients’ outcomes. Until very recently, the absence of a standardized circRNA nomenclature severely complicated the communication of results and replication of circRNA related data. This issue, however, was recently addressed by Chen et al. proposing guidelines for a unified circRNA nomenclature. Now the scientific community should push for the implementation of these guidelines, to benefit the circRNA research field as a whole (Chen et al., 2023). Additionally, best practice standards have been recently put forward by leading experts in the field, highlighting important pitfalls and considerations when designing experiments to ensure reproducibility of findings (Nielsen et al., 2022). Experimental strategies to identify regulatory functions are, currently, primarily based on circRNA depletion or over-expression approaches (Figure 2, step 2), which can give valuable first insights into the modulatory potential of the deregulated circRNAs. Since specific targeting is challenging when it comes to circRNAs, as their sequence is unique exclusively at the BSJ, extra care needs to be taken to avoid simultaneously altering the levels of linear RNA transcripts and splicing. When it comes to over-expression strategies, circRNAs can be either synthetically produced, or transcribed RNA or over-expression plasmids can be transfected into *in vitro* model systems. Next to issues relating to immune activation by the synthetic molecules, these strategies often also lead to delivery/production of linear cognate RNAs and extremely high expression levels of the circRNA of interest, far away from any physiological relevant levels. Thus, the results obtained by such over-expression approaches need to be critically evaluated and, ideally, confirmed with a second strategy.

In general, only few circRNA studies have been able to conclusively demonstrate the molecular action of function of the identified DE circRNA, but merely provide a description of the cellular effects of the modulation of their expression. In order to move the field forward, it will be necessary to thoroughly investigate the molecular mechanisms of action of the candidate circRNAs across different disorders (Figure 2, step 3) to objectively assess their true potential as druggable target, therapeutic agent or biomarker. Once these goals have been reached for a specific candidate circRNA, additional challenges need to be overcome. In case the expression of a candidate circRNA is beneficial and counteracts pathophysiological processes, then the circRNA would need to be overexpressed. Should, instead, a reduction of a candidate circRNA demonstrate desirable effects, knock-down or knock-out approaches would be needed. In either case, all the considerations for overexpression and knock-down strategies discussed above, need to be addressed for a successful translation into the clinics. Independently of a desired up-or downregulation, still, the best approach for reaching the brain needs to be identified. This requires a successful crossing of the blood-brain barrier of a therapeutic agent or small molecule, intracranial injections or intrathecal administration, the targeting of a specific brain region or cell type exclusively, while leaving surrounding areas/cells unaffected. Further limitations are arising from the potential toxicity of all viral and non viral approaches, which may either integrate into the host genome and cause insertional mutagenesis or cause inflammation in the brain. Only when all these hurdles are overcome, then, eventually, circRNAs may benefit patients affected by these complex brain disorders for real (Figure 2, step 4), a milestone that has yet to be achieved by this young research field.
